# Functional Capacity in Congenital Heart Disease: A Systematic Review and Meta-Analysis

**DOI:** 10.5935/abc.20170125

**Published:** 2017-10

**Authors:** Camila Wohlgemuth Schaan, Aline Chagastelles Pinto de Macedo, Graciele Sbruzzi, Daniel Umpierre, Beatriz D. Schaan, Lucia Campos Pellanda

**Affiliations:** 1 Instituto de Cardiologia - Fundação Universitária de Cardiologia, Porto Alegre, RS - Brazil; 2 Hospital de Clínicas de Porto Alegre, Porto Alegre, RS - Brazil; 3 Universidade Federal de Ciências da Saúde de Porto Alegre (UFCSPA), Porto Alegre, RS - Brazil; 4 Universidade Federal do Rio Grande do Sul (UFRGS), Porto Alegre, RS - Brazil

**Keywords:** Heart Defects, Congenital, Child, Adolescent, Exercise Tolerance, Review, Meta-Analysis

## Abstract

**Background:**

Children and adolescents with congenital heart disease often have alterations in their exercise capacity that can be evaluated by various functional testing.

**Objective:**

To evaluate the functional capacity of children and adolescents with congenital heart disease (CHD) with systematic review and meta-analyses.

**Methods:**

The review included observational studies, data from the first evaluation of randomized clinical trials or observational follow-up periods after clinical trials which evaluated functional capacity by cardiopulmonary exercise test, stress testing, six-minute walk test or step test, in children and adolescents with CHD, aged between six and 18 years, and comparisons with healthy controls in the same age group. The quantitative assessment was performed by meta-analysis, by comparing the maximal oxygen consumption (VO_2_max) of children and adolescents with CHD and respective control groups.

**Results:**

Twenty-five of 2.683 studies identified in the search met the inclusion criteria. The VO_2_max measurement showed that patients with CHD have a decrease of 9.31 ml/Kg/min (95% CI. -12.48 to -6.13; I^2^, 94.3%, P for heterogeneity < 0.001) compared with the control group. The meta-analysis of the data of maximum heart rate (HR) reached during cardiopulmonary test and stress testing, retrieved from 18 studies, showed a HR value of -15.14 bpm (95% CI. -20.97 to -9.31; I^2^, 94.3%, P for heterogeneity < 0.001) compared with the control group.

**Conclusion:**

Children and adolescents with CHD have lower VO_2_max and HR compared to controls.

## Introduction

Children with congenital heart disease (CHD) often have a sedentary lifestyle that may reflect both inherent physiological limitations in addition to overprotection of parents.^1^ Such lifestyle pattern is likely to be maintained throughout adulthood, which can result in increased risk for cardiovascular diseases.^1^ In children with restriction for physical activity practice, there is an increased risk for overweight and there is an increasing in overweight (RR, 2.51; 95% CI, 1.24-3.52) and obesity (RR, 6.14; 95% CI, 2.54-8.82) at follow-up.^2^

Functional capacity may indicate cardiovascular, pulmonary or motor dysfunction. In children with chronic disease, maximal oxygen consumption (VO_2_max) can predict adverse outcomes as well as the greater aerobic fitness is associated with a nearly 10% risk reduction for hospitalization of children with cystic fibrosis.^3^ The assessment of functional capacity in patients with heart disease is an important clinical tool for diagnosis, quantification of symptoms, prognosis and evaluation of response to treatment.^4^ Several tests are available to assess functional capacity,^5^ but their use in children and adolescents can give different information than those obtained from adults due to differences in physiological and metabolic responses to stress. Concerning differences in cardiovascular responses, healthy children showed higher chronotropic and lower inotropic responses during maximal effort.^5^ Furthermore, the information of the tests is not standardized in terms of values, which limits the comparison of different studies.

Functional capacity varies according to the type of CHD, surgical outcome, age and gender of the patient. Patients with incomplete repair of heart defects present significant reductions in peak work rate and age-adjusted maximum ventilation as compared with their pairs who undergone complete repair.^6^ Most of the published studies have a small sample size and include children, adolescents and adults, with a large range of age of subjects.^7^ Thus, the present study aimed to systematically review the literature to summarize the functional capacity of children and adolescents diagnosed with CHD, through a meta-analysis of observational studies.

## Methods

### Eligibility criteria

This review included observational studies (cohort, cross-sectional or case-control studies), data from the first evaluation of randomized or non-randomized clinical trials or observational follow-up periods after clinical trials, in which the sample consisted of children and adolescents with CHD, aged between six and 18 years. Other conditions for inclusion of the studies were evaluation of functional capacity by cardiopulmonary exercise test, stress testing, six-minute walk test (6MWT) or step test.

Studies published in English were included. Only studies published after 1980 were considered, since methods for evaluation of functional capacity were not standardized before that period.

### Strategy of search and selection of studies

The following electronic databases were searched in June 2015: MEDLINE (accessed through Pubmed), *Cochrane Central Register of Controlled Trials* (Cochrane CENTRAL) and EMBASE. In addition, references from published studies were also searched manually. Duplicate reports were deleted in the first step of selection of articles. The MeSH terms and entry terms used are presented in Box 1 (Supplementary File).

The titles and abstracts of all articles identified in the search strategy were assessed in duplicate by independent investigators (C.W.S. and A.C.). All abstracts that did not provide sufficient information regarding the inclusion and exclusion criteria were selected for full-text evaluation. In the second phase, the same reviewers independently evaluated these full-text articles and made their selection in accordance with the eligibility criteria. Any disagreements between reviewers were resolved through consensus and, in cases of persistent disagreement, a third reviewer (G.S.) assessed the publications.

### Data extraction

Data were extracted independently by two reviewers (C.W.S and A.C.), using standardized forms comprising methodological characteristics, description of interventions, and outcomes; disagreements were resolved by consensus or by a third reviewer (G.S.).

In order to quantify possible differences on the functional capacity, the primary outcomes were the VO_2_max and the distance walked in the 6MWT. Additionally, maximum heart rate (HR) and other physiological variables taken from the cardiopulmonary exercise test (cardiovascular assessment and gas analyzes with direct measurement of oxygen consumption ), 6MWT and stress testing (cardiovascular assessment, in which symptoms were observed, the behavior of heart rate, blood pressure and electrocardiogram) were also entered into the analyses. Variables extracted from the cardiopulmonary exercise test were the first and second ventilatory thresholds, and from the exercise stress testing we extracted the maximum systolic blood pressure (SBP).

### Assessment of risk of bias

The methodological quality of the studies was assessed by two researchers (C.W.S and A.C.), previously trained and qualified. The *Newcastle-Ottawa Scale* was used for case-control and cohort studies, whereas cross-sectional studies were evaluated with an adaptation of the same scale. The quality score of cohort studies and case-control studies was calculated by the assessment of three components: selection of the study groups (0-4 points), quality of adjustment for confounding (0-2 points) and evaluation of exposure or outcome of interest. The cohort studies evaluation was used for quasi-experimental studies. In the case of cross-sectional studies, the score was calculated in two components: selection of the study groups (0-3 points) and assessment of the outcome of interest (0-4 points). The maximum score could be 9 points for case-control and cohort studies and seven points for cross-sectional studies, representing a high methodological quality.^8^ Disagreement between reviewers were resolved by consensus, and, in cases of persistent disagreement, the assessment was made by a third reviewer (G.S).

### Data analysis

The quantitative assessment of the included studies was performed by meta-analysis, by comparing the VO_2_max in relation to body mass of children and adolescents with CHD and respective control groups without CHD. Combined estimates of effects were generated through the maximum values obtained in the studies reviewed, and are presented as weighted mean differences. Statistical heterogeneity among the results on functional capacity of the studies was assessed by the Cochran's Q test, with significance level of 0.1, and by the inconsistency I^2^ test, in which values above 50% were considered as indicative of high heterogeneity.^9^

The heterogeneity among the studies was explored using two strategies. Initially, each study was individually removed from the meta-analysis in order to verify any particular influence on the results. Second, the influence of age and maximum HR during exercise testing was evaluated by univariate meta-regression, and a threshold of p < 0.05 was used to indicate statistical significance.

The analyses were performed using Stata software version 11.0.

## Results

**Figure 1:**
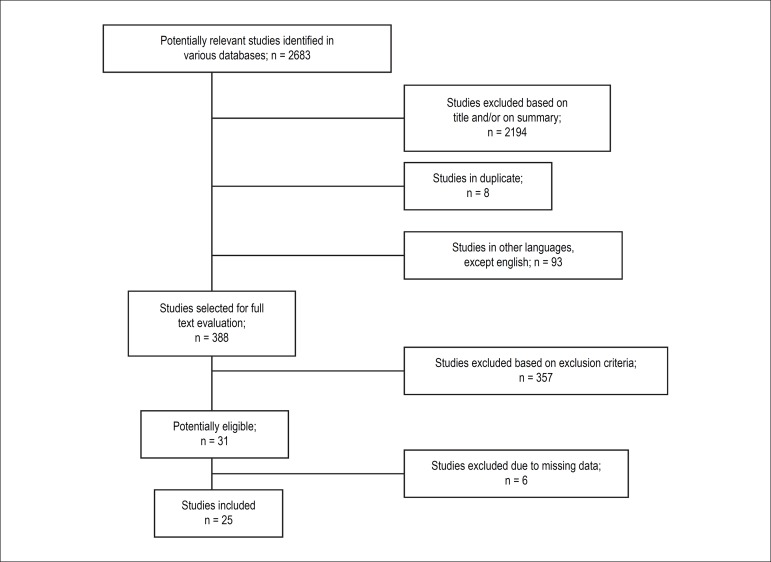
Flowchart of studies evaluated for the meta-analysis.

Twenty-five of the 2.683 studies identified in the search met the criteria of eligibility and were included in the analysis. Figure 1 shows the flow chart of studies of this review. The age of the participants ranged from six to 18 years. Seventeen cross-sectional studies, three quasi-experimental studies and five cross-sectional studies with follow-up were included, with a total of 770 patients with CHD and 754 healthy controls.

The characteristics of the studies are presented in Table 1. Most of the studies investigated children who underwent surgical correction for cyanotic CHD, such as tetralogy of Fallot (T4F), transposition of the great vessels (TGV) and univentricular hearts. Only one study evaluated children that were not submitted previously to surgical correction. Considering the evaluation of functional capacity, 22 studies conducted maximal exercise testing (18 used cardiopulmonary exercise testing and four used the stress testing). In addition to maximal exercise testing, Moalla and collaborators^10^ also performed submaximal test through the 6MWT. Three studies performed submaximal assessment: 1. Hjortdal and collaborators^11^ used the stress test to evaluate the functional capacity up to 1 W/kg on the cycloergometer, and since the participants did not reach their maximal HR with this workload the test was considered as submaximal; 2. Reybrock and collaborators^12^ conducted a cardiopulmonary exercise testing, considering it as a submaximal test, since the assessment was performed to a HR up to 170 bpm; 3. Marcuccio and collaborators^13^ used the cardiopulmonary exercise test, but the maximum HR of the participants was not reached, and the test was therefore considered as submaximal.

**Table 1:** Characteristics of studies included in the systematic review

Study year	Characteristics of participants	Participants (n)	Mean age (SD)	Female	Use of medication	Outcomes and evaluation methods	Values of functional capacity test
**Cross-sectional studies**							
Arvidsson, 2009^24^	Surgically corrected patients (54 patients had undergone biventricular repair), including: AoS, ASD, CoA, DORV, HLV, HRHS, MA, PA, PAPVC, TAPVC, PS, TGV, IVC. NYHA functional class II.	79	9 - 11 years14 - 16 years	37	Not reported	Cardiopulmonary exercise test with cycloergometer; ramp protocol, duration 8-12 minutes and recovery. The patient was instructed to maintain a pedal rate of 60 rpm during the whole exercise test.	Mean and standard deviation of VO_2_max = 42.28 ± 8.8 ml/Kg/min
Giordano, 2003^25^	Surgically corrected aortic coarctation patients. There were 3 end-to-end anastomoses, 10 patch angioplasties, and 7 left subclavian flap repairs.	20	13.7 ± 4.2	8	No antihypertensive medication.	Maximal stress test with treadmill; Bruce protocol. Mean of the time of exercise test = 10.5 ± 2 minutes.	Mean and standard deviation of heart rate = 171 ± 17 bpm
Goldstein, 2011^26^	Participants with Fontan's procedure, excluding patients with pacemaker dependence, severe hypoxemia (oxygen saturation <80% at rest), atrial arrhythmia or several ventricular dysfunction. NYHA functional class I (94%).	51	15 (10.9 -17.8)	20	Not reported	Cardiopulmonary exercise test with treadmill; Bruce protocol.	Median and range of VO_2_max = 28.8 (25.6-33.2)
Grant, 1991^27^	Surgically corrected T4F patients NYHA functional class I.	13	14.1 ± 3	7	Not reported	Cardiopulmonary exercise test with cycloergometer; Godfrey protocol.	Mean and standard deviation of VO_2_max = 28.7 ± 6.6 ml/Kg/min
Groen, 2009^28^	Surgically corrected T4F patients and Fontan's procedure.	13	14 ± 2.8	6	Not reported	Cardiopulmonary exercise test with cycloergometer; Godfrey protocol.	Mean and standard deviation of VO_2_max = 33.7 ± 8.9 ml/Kg/min
Hjortdal, 2008^11^	Participants with Fontan's procedure. NYHA functional class I and II.	14	9.1 ± 5.2	6	Not reported	Submaximal stress test (up to 1 W/kg) with cycloergometer.	Mean and standard deviation of heart rate = 111.5 ± 64.2 bpm
Ishi, 2005^29^	Surgically corrected T4F patients.	26	9.6 ± 3.3	Nonspecific	Not reported	Maximal stress test with cycloergometer; ramp protocol.	Mean and standard deviation of heart rate = 143 ± 11 bpm
Marcuccio, 2012^13^	Surgically corrected T4F patients	21	15 (11-17)	Nonspecific	Not reported	Submaximal stress test with treadmill. Bruce protocol.	Median and range of VO_2_max = 35.8 (23.8-47.8)
Moalla, 2008^30^	Surgically corrected patients including T4F, TGA, IAC, PA. NYHA functional class II and III.	12	13.0 ± 1.2	Nonspecific	Diuretics, cardiotonics, ACE inhibitors.	Cardiopulmonary exercise test with cycloergometer; Wasserman protocol.	Mean and standard deviation of VO_2_max = 30.2 ± 6.1 ml/Kg/min
Mocelin, 1999^31^	Patients corrected for: TGA, IVC, PA, T4F.	35	10.8 ± 2.2	12	Not reported	Cardiopulmonary exercise test with treadmill, constant-load protocol.	Mean and standard deviation of VO_2_max = 42.6 ± 8.6 ml/Kg/min
Page, 1996^15^	Participants with corrected D-TGA.	7	10.4 ± 1.2	4	Not reported	Cardiopulmonary exercise test with treadmill; ramp protocol.	Mean and standard deviation of VO_2_max = 37.6 ± 1.4 ml/Kg/min
Reybrouk, 2000 ^12^	Participants corrected for TGA e T4F.	59	11.2 ± 7.6	24	Not reported	Submaximal exercise test (up to 170 bpm) with treadmill.	Mean of VO_2_max = 40 ml/Kg/min
Sarubbi, 2000^32^	Surgically corrected T4F patients.	41	11.2 ± 3.9	12	No diuretic of cardiotonic medication.	Maximal stress test with cycloergometer.	Mean and standard deviation of heart rate = 167.5 ± 17.4 bpm
Tomassoni, 1991^33^	Surgically corrected T4F patients.	20	9.9 ± 2.8	9	Not reported	Cardiopulmonary exercise test with treadmill; Bruce protocol for >8 years-old and modified Bruce protocol for <8 years-old.	Mean and standard deviation of VO_2_max = 34.1 ± 2.9 ml/Kg/min
Van Beck, 2009^34^	Participants with corrected TGV. NYHA functional class I.	17	12.2 ± 2	5	Not reported	Cardiopulmonary exercise test with cycloergometer; ramp protocol.	Mean and standard deviation of VO_2_max = 41.1 ± 6.6 ml/Kg/min
Muller, 2012^35^	Participants with PS, IVC, IAC, T4F, aortic coarctation, valve stenosis/regurgitation after surgery, Ebstein anomaly, univentricular heart, TGV and TAC. NYHA functional class I and II.	88	12.7 (12.0-13.3)	36	Not reported	Cardiopulmonary exercise test and submaximal exercise test with cycloergometer.	Median and interquartil of VO_2_max = 35.5 (31.3-41.0)
Su, 2013^36^	Participants corrected and non corrected IAC.	50	11.2 ± 3.5	31	Not reported	Cardiopulmonary exercise test with treadmill, Bruce protocol.	Mean and standard deviation of VO_2_max = 31.8 ± 6.8ml/Kg/min
**Quasi-experimental studies**							
Amiard, 2008^37^	Surgically corrected patients including: single ventricle and PA, PA with intact sept, T4F, TGV, IAC.	23	15 ± 1.4	10	ACE inhibitor; diuretics, anticoagulants, cardiotonics, imunosuppressors.	Cardiopulmonary exercise test with cycloergometer; Wasserman protocol.	Mean and standard deviation of VO_2_max = 34.4 ± 10.9ml/Kg/min
Moalla, 2005^10^	Participants surgically corrected for: T4F, TGA, IAC, PA. Functional class NYHA II and III.	17	12.9 ± 0.3	Nonspecific	Diuretics, cardiotonics, ACE inhibitor, except for beta-blocker.	Cardiopulmonary exercise test with cycloergometer; Wasserman protocol. Submaximal test with 6MWT.	Mean and standard deviation of VO_2_max = 28.9 ± 1.7ml/Kg/min
Rutenberg, 1983^38^	Participants corrected for TGA, T4F, valve and aorta diseases.	24	12.8 ± 3.4	8	Not reported	Cardiopulmonary exercise test with treadmill; Bruce protocol.	Mean and standard deviation of VO_2_max = 39.3 ± 8.8ml/Kg/min
**Cross-sectional studies with follow-up**							
Binkhorst, 2008^39^	Participants with corrected and non‑corrected IVC.	27 (13 post-correction IVC and 14 non-corrected), three were excluded from the analysis of functional capacity.	Corrected group = 13 ± 2.5Non-corrected group = 12.5 ± 3	Corrected group = 6Non-corrected group = 8	Not reported	Cardiopulmonary exercise test with cycloergometer, ramp protocol.	Mean and standard deviation of VO_2_max = 45.5 ± 29.2 ml/Kg/min
Carvalho, 1992^40^	Surgically corrected T4F patients.	12	11.3 ± 2.7	Nonspecific	Not reported	Cardiopulmonary exercise test with treadmill; Bruce protocol.	Mean and standard deviation of VO_2_max = 48.0 ± 8.8 ml/Kg/min
Hovels-Gurich, 2003^41^	Surgically corrected TGA patients. NYHA functional class I.	56	10.5 ± 1.6	13	Not reported	Maximal stress test with treadmill; Bruce protocol.	Mean and standard deviation of heart rate = 191.1 ± 10.0 bpm
Musewe, 1988^42^	Surgically corrected TGA patients. NYHA class I.	18	12.8 ± 1.6	7	Not reported	Cardiopulmonary exercise test with cycloergometer; Jones and Campbell protocol.	Mean and standard deviation of VO_2_max = 31.0 ± 7.0 ml/Kg/min
Pfamatter, 2002 ^14^	Participants with corrected IAC.	14	11.4 (6.8 - 16.1)	9	Not reported	Cardiopulmonary exercise test with treadmill; ramp protocol.	Mean and standard deviation of VO_2_max = 37.8 ± 14.8 ml/Kg/min

AoS: aortic stenosis; ASD: atrioventricular septal defect; DORV: double outlet right ventricle; HLV: hypoplastic left ventricle; HRHS: hypoplastic right heart syndrome; MA: mitral atresia; PA: pulmonary atresia; PAPVC: partial anomalous pulmonary venous connection; TAPVC: total anomalous pulmonary venous connection; PS: pulmonary stenosis; TGA: transposition of the great arteries; IVC: interventricular communication; T4F: tetralogy of Fallot; IAC: interatrial communication; TAC: truncus arteriosus communis; ACE: angiotensin-converting-enzyme; HR: heart rate; NYHA: New York Heart Association; 6MWT: six minute walk test. VO_2_max: maximum oxygen consumption

The methodological quality of the cohort studies ranged from two to seven points, with an average of 6.0 ± 1.8 points. For cross-sectional studies, the score varied from three to seven points, with an average of 5.4 ± 1.0. The cohort study with lowest score (by Pfammater et al.^14^ did not describe the origin of the cohort, the methods for assessing the outcome of interest, and how losses were controlled. Among cross-sectional studies, the publication by Page et al.^15^ had only three points, since it did not present non-response rates and did not inform on the representativeness of the sample, origin of the control group and situation of this group (whether it was disease-free). Among quasi-experimental studies, two had four points and one received five points.

**Figure 2:**
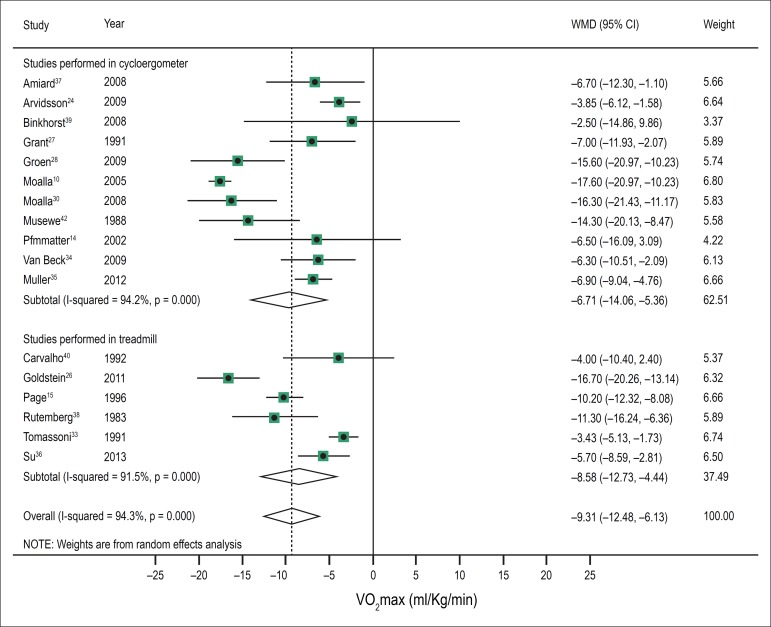
Meta-analysis of maximum oxygen consumption (VO_2_max) in children and adolescents with CHD and in controls, as evaluated on cycloergometer or on treadmill.

In the meta-analysis including 17 studies that conducted cardiopulmonary exercise tests with measurement of VO_2_max, it was 9.31 ml/kg/min lower in patients with CHD (95% CI, -12.48 to -6.13; I^2^, 94.3%, P for heterogeneity < 0.001), as compared with the control group. As shown in Figure 2, studies were stratified according to the type of ergometer used for the maximal test. Eleven studies used the cycloergometer. In these studies, the difference between VO_2_max in the CHD group and the control group was -9.71 ml/Kg/min (95% CI -14.06 to -5.36; I^2^ = 94.2%, P for heterogeneity < 0.00001). Considering the six studies that used the treadmill, the difference between VO_2_max in the CHD group and the control group was -8.58 ml/Kg/min (95% CI -12.73 to -4.44; I^2^ 91.5%, P for heterogeneity < 0.00001).

The meta-analysis on the anaerobic threshold included six studies, showing that the CHD group presented an anaerobic threshold of -4.27 mL/kg/minute (95% CI, -10.84 to 2.31; I^2^, 97.6%, P for heterogeneity < 0.001) as compared with the control group.

**Figure 3:**
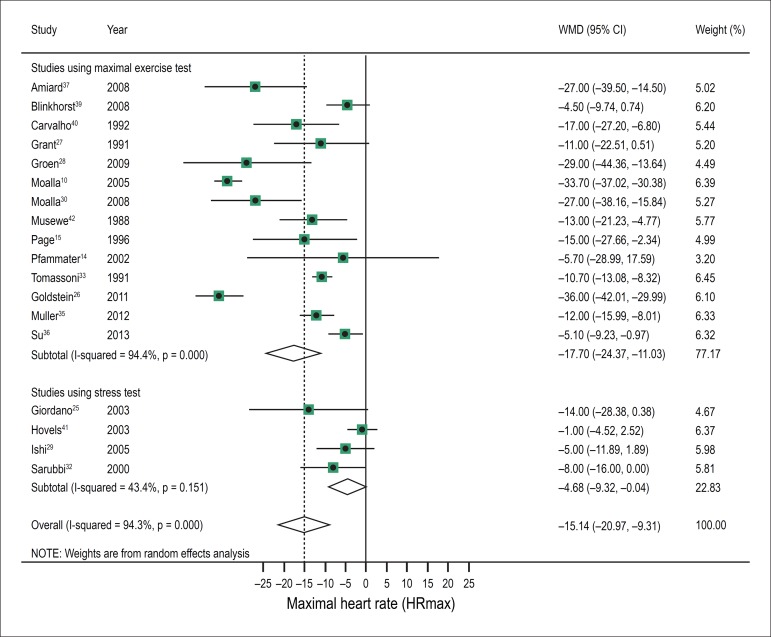
Meta-analysis of maximal heart rate (HRmax) in children and adolescents with CHD and in controls, as evaluated in studies using maximum stress testing and studies using stress test.

Figure 3 shows the meta-analysis of the maximum HR reached during cardiopulmonary exercise test and stress testing, retrieved from 18 studies. The CHD group presented HR of -15.14 bpm (95% CI, -20.97 to -9.31; I^2^, 94.3%, P for heterogeneity < 0.001) as compared with the control group.

Considering the variable HR according to the type of test, 14 studies evaluated maximal HR though the exercise test. In these studies, the CHD group showed a difference of -17.70bpm (95% CI -24.37 to -11.03; I^2^, 94.4%, P for heterogeneity < 0.00001) in relation to the control group. In the four studies that used stress test for evaluation, all presented data as maximal HR. Meta-analysis of these studies showed that the CHD group had a lower HR when compared to the control group (difference -4.68bpm (95% CI -9.32 to -0.04; I^2^, 43.4%, P for heterogeneity = 0.15) (Figure 3).

**Figure 4:**
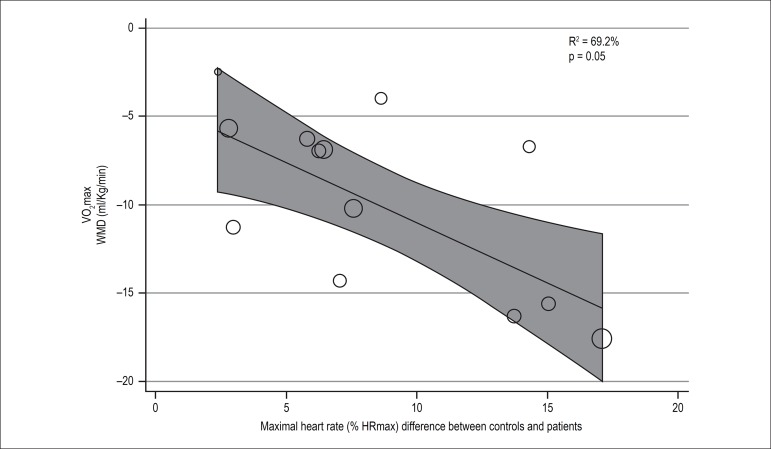
Association between maximum oxygen consumption (VO_2_max) with maximal heart rate (% HR) difference between groups during the maximal exercise test. WMD: weighted mean differences.

The meta-regression showed that the age (n = 16) was not associated with the heterogeneity observed in VO_2_max (R^2^ = 18.43%. p = 0.09). Maximum HR (n = 13), however, had a significant influence on the heterogeneity observed in VO_2_max (R^2^ = 69.20%. p = 0.005), as shown in Figure 4. An inverse relationship is por was between the chronotropic deficit and VO_2_max (β = -0.688; p = 0.005).

Since only one study evaluated functional capacity through the 6MWT, distance walked could not be analyzed. None of the included studies used the step test for evaluation of functional capacity.

## Discussion

This systematic review with meta-analysis of observational studies showed that children and adolescents with CHD present a decrease in functional capacity and in the anaerobic threshold during an exercise maximal test as compared with healthy individuals of the same age group, even when treated. In addition, children and adolescents with CHD have a chronotropic deficit that explained 69.20% of the VO_2_max variance observed among the 13 studies analyzed.

Maximal oxygen consumption (VO_2_max) has been widely used as gold standard for evaluation of functional capacity in healthy or ill individuals. There is a difference in cardiorespiratory responses between adults and children.^5^ The anatomically smaller heart size in children results in lower venous return, and therefore lower cardiac output, which in turn results in lower VO_2_max when compared with adults. Therefore, the most important compensatory mechanism for children is through the increase in HR.^16^ During exercise, the systolic volume increases around 20% in a normal heart, and the further increase in the cardiac output is due to an increase in HR.^17^ Although expected, the information that children and adolescents with CHD in fact have lower functional capacity than their peers, even after corrective surgery, is first summarized in the present meta-analysis.

Individuals with CHD have insufficient chronotropic response, which leads to a decreased maximum HR, consequently reducing the VO_2_max in this population.^17^ Fedriksen et al.^7^ investigated children between eight and 17 years of age with several types of CHD and observed that those aged 10 to 13 years with obstruction of the left ventricular output presented oxygen consumption values above that of those with TGV or T4F. Children with T4F had a natural development of the capacity for physical exercise, which was however lower than that of healthy children; children with TGV showed a decline of VO_2_ between the ages of 12 and 13 years, probably due to a reduction of right ventricular function.^7^ In the present meta-analysis, maximum HR was diminished in 15.14 bpm in the CHD group as compared to the control group. This chronotropic incompetence implies an inability to increase the HR in response to metabolic demand.^18^ The activity of the sympathetic and parasympathetic nervous system, which plays an important role in the modulation of HR during exercise, can be affected by ischemia and/or denervation resulting from surgical procedure or, in cases of cyanotic CHD, by chronic hypoxemia.^19^ Ohuchi et al.^20^ observed that both SBP at rest or during peak exercise and HR variability were lower in the group of children with univentricular hearts compared with healthy controls,^20^ which supports this hypothesis, that the HR directly influences the VO_2_max.

The anaerobic threshold, defined as the maximum intensity of exercise performed by an individual using aerobic metabolism, is inversely related to age.^21^ In a study with 17 children with complex CHD, evaluated by cardiopulmonary exercise testing, Ohuchi et al.^22^ observed that the anaerobic threshold was lower in these children as compared with the control group.^22^ In addition, Paridon et al.^23^ also used cardiopulmonary exercise test to assess 411 children who undergone Fontan procedure showing normal maximal oxygen consumption in 28% of the sample. Maximal oxygen consumption (VO_2_max) within the normal range was observed in only 28% of the sample. However, the anaerobic threshold was in the normal predicted range in most individuals (63%), suggesting that this population with univentricular hearts could tolerate a high level of submaximal and non-maximal activity.^23^

Most studies showed high methodological quality in the evaluation of both exposure and outcome variables. Cross-sectional studies described more detailed evaluations regarding these variables when compared to cohort studies.

The main study limitation derive from that most studies included patients with different types of heart disease, and used different types of evaluation protocols with heterogeneity of ergometers for functional capacity evaluation, even if these are standardized in the literature. Thus, studies showed important differences in relation to these methodological aspects, although all have fulfilled the inclusion criteria for this meta-analysis. High heterogeneity observed in the meta-analyses partially reflects such methodological aspects, and we therefore explored it by using meta-regression analyses for factors of interest. In addition, the heterogeneous nature of the congenital heart lesions may also limit wide exploration of studies in this field, since many lesions have different pathophysiological behaviors and a broad spectrum of severity. In this context, it is important to systematically review all the available information in order to establish more detailed and useful evidence for this specific group.

## Conclusion

The presence of CHD in children and adolescents is associated with lower functional capacity than in healthy controls, measured by VO_2_max in cardiopulmonary exercise testing, being influenced by the impaired chronotropic response observed in this population, and not by age. In addition, a lower ventilatory threshold was observed in the same group, suggesting a lower ability to perform aerobic exercise and consequently tolerate lower exercise loads when comparing to healthy controls of the same age.

## References

[1] Dulfer K, Helbing WA, Duppen N, Utens EM (2014). Associations between exercise capacity, physical activity, and
psychosocial functioning in children with congenital heart disease: a
systematic review. Eur J Prev Cardiol.

[2] Stefan MA, Hopman WM, Smythe JF (2005). Effect of activity restriction owing to heart disease on
obesity. Arch Pediatr Adolesc Med.

[3] Perez M, Groeneveld IF, Santana-Sosa E, Fiuza-Luces C, Gonzalez-Saiz L, Villa-Asensi JR (2014). Aerobic fitness is associated with lower risk of hospitalization
in children with cystic fibrosis. Pediatr Pulmonol.

[4] Wright DJ, Tan LB (1999). The role of exercise testing in the evaluation and management of
heart failure. Postgrad Med J.

[5] Prado DM, Braga AM, Rondon MU, Azevedo LF, Matos LD, Negrao CE (2010). Cardiorespiratory responses during progressive maximal exercise
test in healthy children. Arq Bras Cardiol.

[6] Rosenblum O, Katz U, Reuveny R, Williams CA, Dubnov-Raz G (2015). Exercise Performance in children and young adults after complete
and incomplete repair of congenital heart disease. Pediatr Cardiol.

[7] Fredriksen PM, Ingjer F, Nystad W, Thaulow E (1999). A comparison of VO2(peak) between patients with congenital heart
disease and healthy subjects, all aged 8-17 years. Eur J Appl Physiol Occup Physiol.

[8] Wells GA, Shea B, O'Connell D, Peterson J, Welch V, Losos M The Newcastle-Ottawa Scale (NOS) for assessing the quality of
nonrandomised studies in meta-analyses.

[9] Higgins JP, Thompson SG, Deeks JJ, Altman DG (2003). Measuring inconsistency in meta-analyses. BMJ.

[10] Moalla W, Gauthier R, Maingourd Y, Ahmaidi S (2005). Six-minute walking test to assess exercise tolerance and
cardiorespiratory responses during training program in children with
congenital heart disease. Int J Sports Med.

[11] Hjortdal VE, Christensen TD, Larsen SH, Emmertsen K, Pedersen EM (2008). Caval blood flow during supine exercise in normal and Fontan
patients. Ann Thorac Surg.

[12] Reybrouck T, Mertens L, Brusselle S, Weymans M, Eyskens B, Defoor J (2000). Oxygen uptake versus exercise intensity: a new concept in
assessing cardiovascular exercise function in patients with congenital heart
disease. Heart.

[13] Marcuccio E, Arora G, Quivers E, Yurchak MK, McCaffrey F (2012). Noninvasive measurement of cardiac output during exercise in
children with tetralogy of Fallot. Pediatr Cardiol.

[14] Pfammatter JP, Zanolari M, Schibler A (2002). Cardiopulmonary exercise parameters in children with atrial
septal defect and increased pulmonary blood flow: short-term effects of
defect closure. Acta Paediatr.

[15] Page E, Perrault H, Flore P, Rossignol AM, Pironneau S, Rocca C (1996). Cardiac output response to dynamic exercise after atrial switch
repair for transposition of the great arteries. Am J Cardiol.

[16] Turley KR, Wilmore JH (1997). Cardiovascular responses to treadmill and cycle ergometer
exercise in children and adults. J Appl Physiol (1985).

[17] Amiard V, Jullien H, Nassif D, Maingourd Y, Ahmaidi S (2007). Relationship between dyspnea increase and ventilatory gas
exchange thresholds during exercise in children with surgically corrected
heart impairment. Int J Sports Med.

[18] Reybrouck T, Vangesselen S, Gewillig M (2009). Impaired chronotropic response to exercise in children with
repaired cyanotic congenital heart disease. Acta Cardiol.

[19] Massin MM, Dessy H, Malekzadeh-Milani SG, Khaldi K, Topac B, Edelman R (2009). Chronotropic impairment after surgical or percutaneous closure of
atrial septal defect. Catheter Cardiovasc Interv.

[20] Ohuchi H, Hasegawa S, Yasuda K, Yamada O, Ono Y, Echigo S (2001). Severely impaired cardiac autonomic nervous activity after the
Fontan operation. Circulation.

[21] Reybrouck T, Weymans M, Stijns H, Knops J, van der Hauwaert L (1985). Ventilatory anaerobic threshold in healthy children: age and sex
differences. Eur J Appl Physiol Occup Physiol.

[22] Ohuchi H, Nakajima T, Kawade M, Matsuda M, Kamiya T (1996). Measurement and validity of the ventilatory threshold in patients
with congenital heart disease. Pediatr Cardiol.

[23] Paridon SM, Mitchell PD, Colan SD, Williams RV, Blaufox A, Li JS (2008). A cross-sectional study of exercise performance during the first
2 decades of life after the Fontan operation. J Am Coll Cardiol.

[24] Arvidsson D, Slinde F, Hulthen L, Sunnegardh J (2009). Physical activity, sports participation and aerobic fitness in
children who have undergone surgery for congenital heart
defects. Acta Paediatr.

[25] Giordano U, Giannico S, Turchetta A, Hammad F, Calzolari F, Calzolari A (2005). The influence of different surgical procedures on hypertension
after repair of coarctation. Cardiol Young.

[26] Goldstein BH, Golbus JR, Sandelin AM, Warnke N, Gooding L, King KK (2011). Usefulness of peripheral vascular function to predict functional
health status in patients with Fontan circulation. Am J Cardiol.

[27] Grant GP, Garofano RP, Mansell AL, Leopold HB, Gersony WM (1991). Ventilatory response to exercise after intracardiac repair of
tetralogy of Fallot. Am Rev Respir Dis.

[28] Groen WG, Hulzebos HJ, Helders PJ, Takken T (2010). Oxygen uptake to work rate slope in children with a heart, lung
or muscle disease. Int J Sports Med.

[29] Ishii H, Harada K, Toyono M, Tamura M, Takada G (2005). Usefulness of exercise-induced changes in plasma levels of brain
natriuretic peptide in predicting right ventricular contractile reserve
after repair of tetralogy of Fallot. Am J Cardiol.

[30] Moalla W, Dupont G, Temfemo A, Maingourd Y, Weston M, Ahmaidi S (2008). Assessment of exercise capacity and respiratory muscle
oxygenation in healthy children and children with congenital heart
diseases. Appl Physiol Nutr Metab.

[31] Mocellin R, Gildein P (1999). Velocity of oxygen uptake response at the onset of exercise: a
comparison between children after cardiac surgery and healthy
boys. Pediatr Cardiol.

[32] Sarubbi B, Pacileo G, Pisacane C, Ducceschi V, Iacono C, Russo MG (2000). Exercise capacity in young patients after total repair of
Tetralogy of Fallot. Pediatr Cardiol.

[33] Tomassoni TL, Galioto FM Jr, Vaccaro P (1991). Cardiopulmonary exercise testing in children following surgery
for tetralogy of Fallot. Am J Dis Child.

[34] van Beek E, Binkhorst M, de Hoog M, de Groot P, van Dijk A, Schokking M (2010). Exercise performance and activity level in children with
transposition of the great arteries treated by the arterial switch
operation. Am J Cardiol.

[35] Muller J, Bohm B, Semsch S, Oberhoffer R, Hess J, Hager A (2013). Currently, children with congenital heart disease are not limited
in their submaximal exercise performance. Eur J Cardiothorac Surg.

[36] Su CT, Sung TY, Lin KL, Wang JL, Yang AL (2013). Lower exercise capacity in children with asymptomatic atrial
septal defect associated with circulatory impairment. Chin J Physiol.

[37] Amiard V, Jullien H, Nassif D, Bach V, Maingourd Y, Ahmaidi S (2008). Effects of home-based training at dyspnea threshold in children
surgically repaired for congenital heart disease. Congenit Heart Dis.

[38] Ruttenberg HD, Adams TD, Orsmond GS, Conlee RK, Fisher AG (1983). Effects of exercise training on aerobic fitness in children after
open heart surgery. Pediatr Cardiol.

[39] Binkhorst M, van de Belt T.de Hoog M.van Dijk A.Schokking M.Hopman M (2008). Exercise capacity and participation of children with a
ventricular septal defect. Am J Cardiol.

[40] Carvalho JS, Shinebourne EA, Busst C, Rigby ML, Redington AN (1992). Exercise capacity after complete repair of tetralogy of Fallot:
deleterious effects of residual pulmonary regurgitation. Br Heart J.

[41] Hovels-Gurich HH, Kunz D, Seghaye M, Miskova M, Messmer BJ, von Bernuth G (2003). Results of exercise testing at a mean age of 10 years after
neonatal arterial switch operation. Acta Paediatr.

[42] Musewe NN, Reisman J, Benson LN, Wilkes D, Levison H, Freedom RM (1988). Cardiopulmonary adaptation at rest and during exercise 10 years
after Mustard atrial repair for transposition of the great
arteries. Circulation.

